# Nanoliposome-Loaded Phenolics from *Nasturtium officinale* Improves Health Parameters in a Colorectal Cancer Mouse Model

**DOI:** 10.3390/ani12243492

**Published:** 2022-12-10

**Authors:** Fatemeh Taghavinia, Fatemeh Teymouri, Fatemeh Farokhrouz, Elahe Hashemi Bagherabad, Sarasadat Farjami, Ehsan Karimi, Ehsan Oskoueian, Hieu Huu Le, Majid Shakeri

**Affiliations:** 1 Department of Biology, Mashhad Branch, Islamic Azad University, Mashhad 917568, Iran; 2 Department of Research and Development, Arka Industrial Cluster, Mashhad 9188944586, Iran; 3 Faculty of Animal Sciences, Vietnam National University of Agriculture, Trau Quy, Hanoi 131004, Vietnam; 4 U.S. National Poultry Research Center, Agricultural Research Service, USDA, Athens, GA 30605, USA

**Keywords:** phytogenic, phytobiotic, health benefits, colon, encapsulation, gene expression

## Abstract

**Simple Summary:**

*Nasturtium officinale* contains a sufficient amount of minerals, vitamins and flavonoid compounds which can act as antioxidants to reduce oxidative damages in tissues. It has been considered as a good additive to cleanse toxins from the body and inhibit the development of cancer cells. The present study attempted to investigate the health benefits of nonencapsulated and nanoliposome-encapsulated phenolic rich fractions obtained from *Nasturtium officinale* on mice induced colorectal cancer. The experiment focused on encapsulation efficiency in improving the effectiveness of plant bioactive compounds. The results showed that phenolic rich fractions improved health condition of mice by improving Caspase 3, Bax, Bcl2, iNOS and SOD genes in the tumor tissue. Furthermore, phenolic rich fractions improved intestinal morphology when the mice were challenged with colorectal cancer. The health benefits observed in this study could be associated with its enhanced intestinal absorption, bioavailability, bioaccessibility and bioactivity. The results of the study concluded that the nanoliposome-encapsulated phenolic rich fractions could be considered as a promising anticancer agent against colorectal cancer.

**Abstract:**

*Nasturtium officinale* contains high amounts of phytochemical compounds that work against oxidative damages leading to improved health conditions in animals as well as humans. The study was performed to investigate the health benefits of nonencapsulated and nanoliposome-encapsulated phenolic rich fractions obtained from *Nasturtium officinale* on mice induced colorectal cancer. The experiment focused on encapsulation efficiency in improving the effectiveness of plant bioactive compounds. Phenolic rich fractions (PRF) were successfully loaded in the nanoliposome structure, a nanometer in size, of spherical shape and with homogeneous dispersion. Induction of colorectal cancer in mice impaired weight gain and feed intake, liver function and structural characteristics of ileum, while the dietary administration of nanoliposome-encapsulated PRF regulated the expression of Caspase 3, Bax, Bcl2, iNOS and SOD genes in the tumor tissue. The addition of nonencapsulated PRF and nanoliposome encapsulated PRF at the concentration of 100 mg TPC/kg BW/day improved the genes expression, although the nanoliposome-encapsulated PRF revealed better health outcomes compared to nonencapsulated PRF. Furthermore, both PRF improved intestinal morphology when the mice were challenged with colorectal cancer. The higher health promoting activity of nanoliposome-encapsulated PRF could be associated with its enhanced intestinal absorption, bioavailability, bioaccessibility and bioactivity. Consequently, the nanoliposome-encapsulated PRF could be considered as a promising anticancer agent against colorectal cancer.

## 1. Introduction

Abnormal proliferation of various types of cells in the body can result in hundreds of different types of cancer, while their responses to therapy vary substantially [1]. One common type of cancer worldwide is colorectal cancer. It is a serious public health issue and one of the important diagnosed cancers. There are several major factors that could cause this cancer including smoking, low intake of vegetables and fruits, alcohol consumption, and overweight and obesity [2,3].

Chemotherapy development has led to a higher survival rate of colorectal cancer patients over the past years. However, resistance to chemotherapy and side effects are still limitations of conventional chemotherapy, and safe and natural alternative remedies are required to mitigate side effects in the long term. In this regard, medicinal plants that possess natural bioactive compounds demonstrated significant apoptogenic and cytotoxic activities against different kinds of cancer. Among these compounds, polyphenols including curcumin, ellagic acid, gallic acid and epigallocatechin-3-gallate offer a wide range of health benefits [4,5] and play a preventive role against colon cancer. One of the more promising natural remedies is *Nasturtium officinale*, which has antioxidant and anticancer properties.

*Nasturtium officinale*, a perennial aquatic weed, belongs to the Brassicaceae family, and is considered as an important medicinal plant due to the presence of various types of bioactive components including phenolic and flavonoids [6]. In the study conducted by Sefidkon et al. [7], the flavonoid compounds in the dichloromethane fraction obtained from leaves and flowers of *Nasturtium officinalis* possessed anticancer effects against breast cancer cells (T47D) and colon cancer cells (HT-29). In another study conducted by Fallah and Ebrahimi [8] the hydroalcoholic extract obtained from *Nasturtium aquaticum* inhibited the growth of breast cancer cells (MDA-MB-231 cells). Similarly, the treatment of lung carcinoma A549 cells with *Nasturtium officinale* extract up-regulated the expression of p53, Bax and caspase-3 genes, confirming its anticancer effects against lung cancer [9]. The study conducted by Mazandarani et al. [10] revealed that phenolic and flavonoid compounds are the major bioactive compounds present in this plant.

Despite the particular anticancer and pharmacological properties of natural compounds, their low bioavailability and selectivity limit their therapeutic application. Therefore, drug delivery systems such as liposomes, nanoemulsions, films and nanoparticles are designed to increase targeting, pharmacokinetics efficacy and cellular uptake of anticancer plant constituents [11]. A study reported that physicochemical properties, drug loading and release of curcumin were enhanced by nanoencapsulation. Selective and potential cytotoxic impacts of curcumin nanoencapsulation against colon cancer were also indicated in this study [12]. Another study compared the potential of gold nanoparticles loaded 99mTc-resveratrol with albumin loaded 99mTc-resveratrol for tumor special delivery. Results indicated that albumin is a premier molecule for the impressive delivery of 99mTc-resveratrol at tumor locations in comparison with gold nanoparticles [13]. Considering the scarcity of investigations on biological properties of *Nasturtium officinale*, the objective of this research was to evaluate the anticancer activity and the molecular mechanism of nanoliposome-encapsulated phenolic rich fractions of *Nasturtium officinale* compared with its un-capsulated extract.

## 2. Materials and Methods

### 2.1. Ethic Statement

All procedures were carried out in accordance with the ethical principles approved by the Islamic Azad University of Mashhad, Iran, with the code of ethics IR.IAU.MSHD.REC.1399.018, November 2020.

### 2.2. Plant Material and Reagents

*Nasturtium officinale* was purchased from a local market located in Mashhad, Iran. Soybean lecithin with purity of 99% was used for the study (Sigma Aldrich, Saint Louis, MO, USA). RNeasy Mini kit and all required reagents were purchased from Qiagen (Hilden, Germany) and Readington Township, (NJ, USA).

### 2.3. Fractionation and Total Phenolic Determination

A grinder mill was used to finely grind the aerial parts to powder form. The determination was done based on two previously published methods [14,15]. The absorbance of the samples was measured at 765 nm. We expressed the results in milligrams of gallic acid equivalents per gram dry weight. Phenolic rich fractions (PRF) are the fractions indicating the highest level of phenolic compounds.

### 2.4. Nanoliposomes Preparation

At 80 °C, 100 mL of hot water was agitated for 2 h with 4 g of lecithin at 300 rpm for 2 h. The PRF was dissolved in ethanol and added to the mixture and shook for 2 h to obtain the final concentration of 2000 ppm. After bath sonication of the suspension at 80% (Sonorex RK100, Germany) for 4 min, a nanoliposome-loaded PRF from *Nasturtium officinale* was used for further analysis.

### 2.5. Characterization of Nanoliposomes

In order to reduce aggregation and to inhibit noise scattering, nanoliposomes-loaded PRF were diluted by water (1:20). The average particle size as well as the zeta potential (stability) of particles were both determined using the dynamic light scattering method. A previously published method was used to measure the total phenolic content of nanoliposomes [15].

### 2.6. Phenolic Profiling of Nanoliposomes

High performance liquid chromatography (RP-HPLC) was used to identify the types of phenolic compounds in the nanoliposomes (Figure S1). The pH was adjusted by adding trifluoroacetic acid concentrated in 33% aqueous solution in deionized water (solvent A) and acetonitrile (solvent B). Equilibration of the column was done using a mixture of 85.5% of solvent A with 15% of solvent B for 15 min. After 50 min, the ratio of solvent B was increased to 85%. Approximately 5 min after starting the experiment (at 55th min), the proportion of solvent B reduced to 15%. A flow rate of 1 mL/min was used for the next analysis after maintaining this ratio for 60 min. Phenolic was detected at 280 nm using an analytical column (Intersil ODS-3 5 μm 4.6 ∗ 150 mm GL Science Inc., Torrance, CA, USA). Syringic acid, gallic acid, caffeic acid, catechin, naringin, vanillic acid, salicylic acid, pyrogallol, cinnamic acid, ellagic acid, chrysin and ferulic acid were the phenolic standards utilized in this study.

### 2.7. Animal Trial

Twenty-four white male Balb/c mice (Razi Vaccine and Serum Research Institute, Mashhad, Iran) with a weight of approximately 25 g were housed in cages. They were divided into individual cages for 7 days where humidity was 58 ± 10% and temperature was 23 ± 1 °C with 12 h light/dark periods to adapt to laboratory conditions. All the mice were inoculated with colon cell line (HT-29 cancer cell) after being divided into 3 groups of 8 mice as describer earlier [16]. The groups were divided into 3 experimental treatments: (T1) induced colorectal cancer; (T2) induced colorectal cancer received 100 mg/kg BW/day phenolics; and (T3) induced colorectal cancer received 100 mg/kg BW/day nanoliposome-loaded phenolics

All mice were monitored daily for their general health and feed consumption. On day 28, pentobarbital-HCL (50 mg/kg, i.p.) was used to euthanize the mice. Blood, liver and tumor tissues were collected immediately after euthanization.

### 2.8. Blood Parameters, Liver Enzymes and Lipid Peroxidation Assay

Blood auto-analyzer (Hitachi 902, Japan) was used to determine the main liver enzymes in the serum including aspartate transaminase (AST), alanine aminotransferase (ALT) and alkaline phosphatase (ALP), as well as blood parameters such as red blood cell (RBC), white blood cell (WBC), monocyte, lymphocyte and neutrophile. Peroxidation of lipids within liver tissue was determined based on a previously published method [10]. Obtained N-butanol absorbance was measured at 532 nm and expressed as percentage malondialdehyde (MDA) changes in comparison to the control.

### 2.9. Histopathology and Morphometric Analyses

Intestines, livers, kidneys, spleens and ileums were removed and washed with the normal saline. Thereafter, buffered formalin (10% formalin in 0.1 M sodium phosphate buffer, pH7) was used to fix them. Then, paraffinizing, slicing and staining the tissues was undertaken in accordance with the hematoxylin/eosin protocol [17]. A 20X magnification was used to look at the histopathological slides under a light microscope. A variety of morphostructural features of the ileum were examined, including villus width, villus height, number of goblet cells and crypt depth [18].

### 2.10. Gene Expression Analysis

A number of main biomarker genes in the tumor were examined in this study, including Bax, caspase 3, inducible nitric oxide synthase (iNOS), Bcl2 and superoxide dismutase (SOD). RNA extracted from fresh frozen samples using RNeasy Mini kit (Qiagen, Hilden, Germany), followed by reverse transcription (Hilden, Germany). SYBR Green PCR Master Mix (Qiagen, Hilden, Germany), were used to perform a comparative real-time PCR (Roche Diagnostics). Amplification of targeted genes was as follows: 5 min at 95 °C, followed by 35 cycles for 30 s at 95 °C, primer annealing for 30 s at 60 and 58 °C for the inflammatory genes and antioxidant genes, respectively, and 30 s in 72 °C for the extension. As a reference gene, β-actin was used as a normalizing gene, and genes’ expressions were compared to the expression of their respective genes in controls [19]. The primer sequences are presented in Table 1.

### 2.11. Statistical Analysis

The results were analysed using the general linear model (GLM) procedure of SAS Version. 9.1 (SAS Institute Inc., Cary, NC, USA)] in a completely randomized design (CRD). Duncan’s multiple range test was used to be compared with means. *p*-values less than 0.05 were considered significant.

## 3. Results

### 3.1. Fractionation and Total Phenolic Determination

The fractionation with different polarity solvents resulted in the extraction of most of the phenolic compounds from leaves of *Nasturtium officinale* in different quantities of which the highest phenolic compounds were found in ethyl acetate fraction (26.1 ± 4.36) followed by n-butanol (16.7 ± 3.29), water (12.6 ± 3.94), chloroform (8.3 ± 2.76) and hexane (7.5 ± 2.63) mg GAE/g DW of extract. Ethyl acetate was found to be the fraction containing the most phenolic compounds and is therefore termed a “phenolic rich fraction (PRF)” for further experiments. According to our earlier unpublished data, phenolic concentration is highest in the ethyl acetate fraction when extracting phenolic compounds with different polarity solvents.

### 3.2. Physicochemical Characteristics of PRF Loaded Nanoliposome

**Figure 1:**
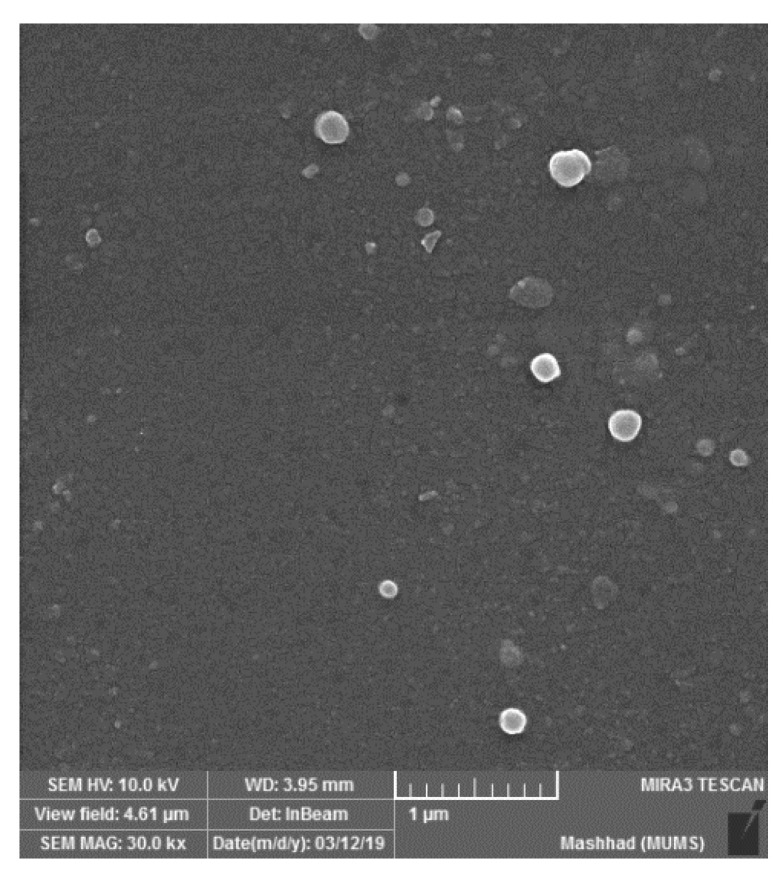
Field emission scanning electron microscopy analysis of nanoliposome loaded by *Nasturtium Officinale*.

An important factor in nanoliposomes’ stability and bioavailability is particle size. The particle size was 189.2 ± 11.68 nm, which was a nanometer-sized particle. Polydispersity index values are reported as 0.29 ± 0.04, which indicates homogeneous dispersion (Table 2). Total phenolic compounds in the nanoliposomes were 10.9 ± 0.12 mg GAE/g DW (Figure 1).

### 3.3. Analysis of Phenolic Compounds

The obtained results indicated that there are various phenolic compounds in the nanoliposomes, with ellagic acid and catechin being the most abundant and significant phenolic compounds with an abundance of 702.5 ± 6.2 and 681.8 ± 4.5 µg/g DW, respectively (Table 3). The analyses were performed in triplicate.

### 3.4. Weight and Feed Intake Alteration

The overall results showed a significant (*p* < 0.05) decrease in the average daily weight gain and feed intake in mice induced with colorectal cancer (T1), while our results indicated that the addition of non-encapsulated PRF and nanoliposome-encapsulated PRF (T2 and T3) significantly improved these parameters (*p* < 0.05). Supplementing phenolic compounds with liposomes increased weight gain and feed intake when compared to supplementing with non-encapsulated phenolic compounds (Table 4).

### 3.5. Analysis of Serum and Blood Biomarkers

**Figure 2:**
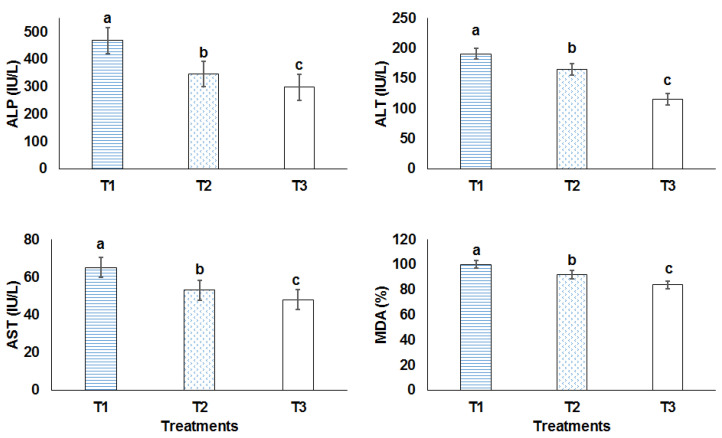
Enzymes and lipid peroxidation changes in liver. T1: induced colorectal cancer; T2: induced colorectal cancer received 100 mg/kg/day phenolics; T3: induced colorectal cancer received 100 mg/kg/day nanoliposome-loaded phenolics. Different letters indicated significant difference (*p* < 0.05).

Hepatocyte damage and liver toxicity are evaluated with liver enzymes (ALP, ALT, and AST) and MDA as histological markers of lipid peroxidation (the analysis was performed in triplicate, Figure 2). Improved health status is reflected in the reduction of liver enzyme production and lipid peroxidation. Furthermore, mice treated with nanoliposome-loaded phenolics and induced with colon cancer increased their liver enzyme production and lipid peroxidation, indicating signs of liver damage. Blood parameters including RBC, WBC, lymph, eosinophils, monocytes and neutrophil are presented in Table 5 The results showed that 100 mg/kg/day of phenolics and nanoliposome-loaded phenolics did not alter the analyzed blood parameters.

### 3.6. Histopathological Examination

**Figure 3:**
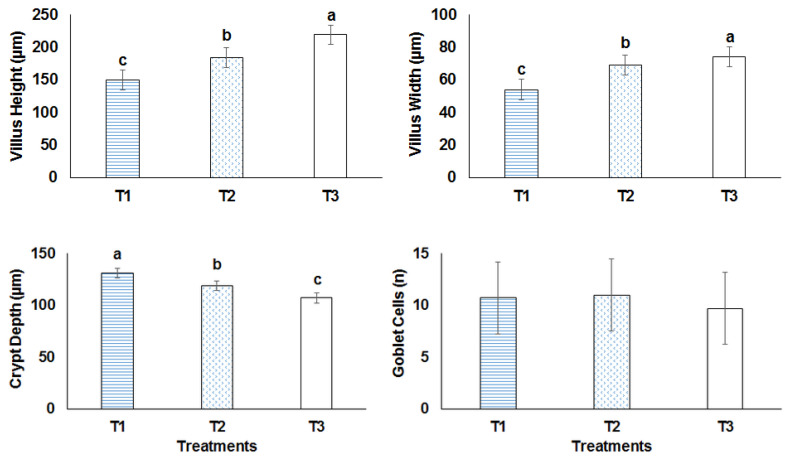
Morphometric analysis of ileum in different treatments. T1: induced colorectal cancer; T2: induced colorectal cancer received 100 mg/kg/day phenolics; T3: induced colorectal cancer received 100 mg/kg/day nanoliposome-loaded phenolics. Different letters indicated significant difference (*p* < 0.05).

**Figure 4:**
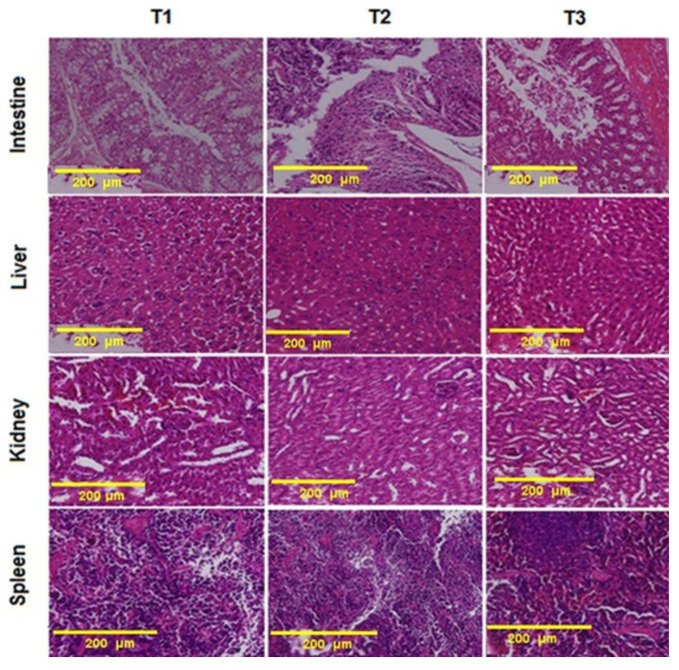
The histopathological changes in the mice intestine, liver, kidney and spleen challenged by colorectal cancer. T1: induced colorectal cancer, T2: induced colorectal cancer received 100 mg/kg/day phenolics, T3: induced colorectal cancer received 100 mg/kg/day nanoliposome-loaded phenolics.

The results indicated that 100 mg/kg/day phenolics and 100 mg/kg/day phenolics-loaded nanoliposomes for 28 days had no histological changes except in the intestine (Figure 3). Morphometric images of villus width, villus height, crypt depth and number of goblet cells in the ileum of mice that received different treatments are presented in Figure 4. The findings showed that villus width and villus height increased whereas the crypt depth decreased significantly (*p* < 0.05) as a result of the oral administration of 100 mg/kg/day phenolics. The number of goblet cells did not show any changes upon different treatments. It highlighted the role of the phenolic compounds in improving the morphostructure of the ileum in different animals including rabbits, pigs, rats and the broiler chickens.

### 3.7. Gene Expression Analysis

Gene profiling pattern of the tumor demonstrated an increase in apoptosis upon causing colon cancer. However, consumption of a dietary supplement of 100 mg/kg/day nanoliposome-loaded phenolics has significantly (*p* ≤ 0.05) regulated the gene expression (Table 6).

## 4. Discussion

The main findings of the study indicated that the addition of nonencapsulated PRF and nanoliposome encapsulated PRF at the concentration of 100 mg TPC/kg BW/day improved mice weight gain, feed intake, liver function and the structural characteristics of ileum. Additionally, they regulated the expression of apoptotic genes (Caspase 3, Bax, Bcl2, iNOS, SOD) in the tumor, although the nanoliposome-encapsulated PRF were revealed to be of higher potential, compared to nonencapsulated PRF, in improving the health parameters.

The nanoencapsulation with nanoliposomes was reported in previous studies to improve the effectiveness of colon cancer therapy and decrease the side effects [20,21]. In order to have stable and bioavailable nanoliposomes, the size has to be considered, (189.2 ± 11.68 in current study). It affects various aspects, including encapsulation efficacy, drug release profile and uptake of nanoparticles by targeted cells [22]. Furthermore, the polydispersity index is a parameter indicating homogeneous dispersion of nanoparticles and its acceptable amount was reported to be less than 0.7, which was 0.29 ± 0.04 for the current study, indicating a higher stability compared to bigger sizes [23,24].

In terms of *Nasturtium officinale* as a medicinal plant, it changes the blood’s antioxidant status with cholesterolemia, decreases oxidative stress, increases antioxidant capacity through the elevation of glutathione and increases the activities of catalase and superoxide dismutase in liver [25]. On the other hand, it reduces hepatic MDA along with glutathione reductase and glutathione peroxidase [25]. In addition, *Nasturtium officinale* extract with gallic acid and catechin as phenolic and flavonoid contents could exert its antioxidant activities through metal chelating and the direct trapping of free radicals [26,27]. Our results have also indicated that the phenolic compounds of this plant reduced oxidative stress in tissues that resulted from induced colorectal cancer in mice by increasing SOD gene expression as an antioxidant agent and also decreased MDA gene expression. MDA is one of the most noxious products of lipid peroxidation and its determination can be used to estimate the severity of oxidative stress [28,29]. Moreover, alteration of some other genes like Bax, Caspase3, Bcl-2 and iNOS affect the cancer’s progression. Up-regulation of caspase3 and Bax gene expression in colorectal cancer cells can induce apoptosis and inhibit cancer development [30,31]. However, down-regulation of Bcl-2 inhibits cell apoptosis as its expression was indicated to be associated with pathological parameters and better prognosis. Cellular fate can be determined through the balance among antiapoptotic Bcl-2 and proapoptotic Bax in cells [32]. A report indicated the involvement of iNOS in inflammation, tumor initiation and development [33]. Based on our results, treatments with the phenolics and nanoliposome-loaded phenolics could inhibit cell progression and inflammation by increasing the expression of apoptotic genes (Bax, Caspase3) and decreasing the antiapoptotic and inflammatory associated gene of Bcl-2 and iNOS in the tumor tissue.

Liver enzymes’ (ALT, ALP, and AST) alteration could be an indication of liver injury; they release in the blood after an hepatic injury [34]. Phenolic and phenolics-loaded nanoliposome reduced these biomarkers but the latter one was more beneficial in terms of this reduction. The hepatoprotective function of *Nasturtium officinale* was proved in previous studies. For instance, a study illustrated that 500 mg/kg of this plant reduced the level of AST and ALT in the rats with hepatotoxicity, which was induced by cyclophosphamide [35]. It is suggested that most of its hepatoprotective activities are due to its antioxidant activity, the existence of rich essential nutrients and secondary metabolites with health promoting features like phenolics and glucosinolates [36,37]. Phenolics and phenolic-loaded nanoliposomes cannot alter the blood parameters including RBC and WBC. One study showed that the supplementation of 1% *Nasturtium officinale* extract per kg food might improve some immunological and hematological parameters in fish, such as hemoglobin concentration, hemoglobin, complement activities and lysosome. However, unlike our obtained results, it did not change the RBC and WBC count [38].

The histopathology of liver, kidney and spleen did not indicate any particular changes after treatments. The intestine was improved after the treatments. Villus height increased, which is the characteristic of good functionality and integral intestinal health. It has been reported that herbal feed supplementations could increase villus height and decrease crypt depth of the small intestines of broiler chickens [39]. Intestinal morphology improvement should be associated with phenolic substances, which affect the intestinal cells’ proliferation directly [40].

## 5. Conclusions

The current experiment focused on encapsulation efficiency in improving the effectiveness of plant bioactive compounds. Phenolic enrich fraction-loaded nano-liposomal of *Nasturtium officinale* with the size of 189.2 nm and PDI of 0.29 consist of different types of natural bioactive compounds including gallic acid, salicylic acid, syringic acid, catechin and ellagic acid. It significantly developed health parameters by improving Caspase 3, Bax, Bcl2, iNOS and SOD genes in the tumor tissue. Furthermore, PRF improved intestinal morphology when the mice were challenged with colorectal cancer. Nanoliposome-encapsulated PRF alleviated cancer induction symptoms more effectively, indicating probable benefits to the treatment of colorectal cancer.

## Figures and Tables

**Table 1:** Primer sets used in this study.

Gene	Forward (5′ to 3′)	Reverse (5′ to 3′)
Caspase 3	CACAATAGCACCCATCCG	GGGACATCAGTCGCTTCA-3
Bax	AATGCCCGTTCATCTCAG	GGGACATCAGTCGCTTCA
Bcl2	GGCACTGTCTTGACCCAC	TCATAAACCCTGCTTGCTG
iNOS	CACCTTGGAGTTCACCCAGT	ACCACTCGTACTTGGGATGC
SOD	GAGACCTGGGCAATGTGACT	GTTTACTGCGCAATCCCAAT
β-actin ^1^	CCTGAACCCTAAGGCCAACC	CAGCTGTGGTGGTGAAGCTG

^1^ Housekeeping gene.

**Table 2:** Physical characteristics of nanoliposome-loaded *Nasturtium officinale* phenolic compounds.

Particle Size (nm)	Polydispersity Index (PDI)	Zeta Potential (mV)
189.2 ± 11.68	0.29 ± 0.04	−58.2 ± 6.82

**Table 3:** Phenolic compounds in the nanoliposome-loaded *Nasturtium officinale* phenolic rich fractions.

Phenolic Compounds Contents (µg/g DW)				
GA ^1^	SA	SY	CA	EA
438.3 ± 2.7	206.2 ± 5.1	287.3 ± 1.0	681.8 ± 4.5	702.5 ± 6.2

^1^ GA: gallic acid; SA: salicylic acid; SY: syringic acid; CA: catechin; EA: ellagic acid.

**Table 4:** Average body weight and feed intake of mice when supplemented with different treatment.

Average ^1^	T1	T2	T3	SEM
Average daily weight gain (mg)	39.1 ^c^	46.5 ^b^	51.9 ^a^	2.13
Average daily feed intake (g)	1.46 ^c^	1.54 ^b^	1.63 ^a^	0.03

^1^ T1: induced colorectal cancer; T2: induced colorectal cancer received 100 mg/kg/day phenolics; T3: induced colorectal cancer received 100 mg/kg/day nanoliposome-loaded phenolics. Different letters in the same row indicated significant difference (*p* < 0.05).

**Table 5:** Blood parameters changes of mice when supplemented with different diets.

	RBC ^1^ (10^6^/µL)	WBC (10^3^/µL)	NEU (%)	Lymph (%)	Mno (%)	Eo (%)
T1 ^2^	7.18	4.9	35	58	5	2
T2	7.44	5.9	36	57	4	3
T3	7.56	6.1	35	61	3	1
SEM	0.92	7.63	2.48	2.75	0.48	0.61

^1^ RBC: red blood cells; WBC: white blood cells; NEU: neutrophil; Mno: monocytes; Eo: eosinophils. ^2^ T1: induced colorectal cancer; T2: induced colorectal cancer received 100 mg/kg/day phenolics; T3: induced colorectal cancer received 100 mg/kg/day nanoliposome-loaded phenolics.

**Table 6:** Alterations in the expression of apoptosis related genes in mice colon tissue under different treatments.

	Gene Expression (Fold Changes)			SEM
Genes	T1 ^1^	T2	T3	
Up-regulated genes				
Caspase-3	1.0 ^c^	1.7 ^b^	2.4 ^a^	0.08
Bax	1.0 ^c^	2.9 ^b^	3.2 ^a^	0.11
SOD	1.0 ^c^	1.8 ^b^	2.5 ^a^	0.09
Down-regulated genes				
Bcl2	1.0 ^c^	1.6 ^b^	2.2 ^a^	0.12
iNOS	1.0 ^c^	1.9 ^b^	2.7 ^a^	0.15

^1^ T1: induced colorectal cancer; T2: induced colorectal cancer received 100 mg/kg/day phenolics; T3: induced colorectal cancer received 100 mg/kg/day nanoliposome-loaded phenolics. Different letters in the same raw indicated significant difference (*p* < 0.05).

## Data Availability

The data presented in this study are available on request from the corresponding author.

## Supplementary Materials

The following are available online at https://www.mdpi.com/article/10.3390/ani12243492/s1, Figure S1: The RP-HPLC spectra of different bioactive compounds in nanoliposome-encapsulated phenolic rich fraction of Nasturtium officinale (ND = Not detected).
