# Supplementary material for: Nanoliposome-Loaded Phenolics from Nasturtium officinale Improves Health Parameters in a Colorectal Cancer Mouse Model
Source: Animals (Basel). 2022 Dec 10;12(24):3492. doi: 10.3390/ani12243492 (PMC9774266; doi:10.3390/ani12243492)
Supplement: Supplementary file 1 [file animals-12-03492-s001.zip › animals-2005672-supplementary.pdf]

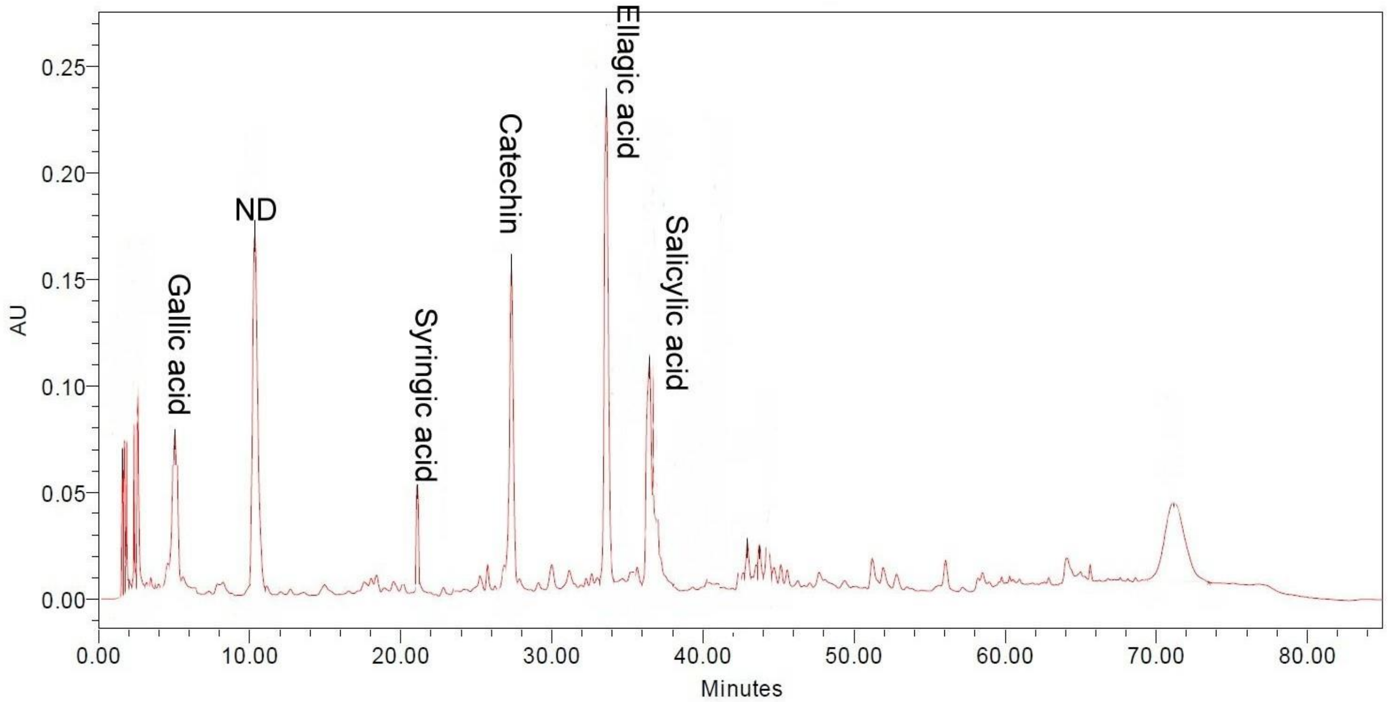

Figure S1. The RP-HPLC spectra of different bioactive compounds in nanoliposome-encapsulated phenolic rich fraction of *Nasturtium officinale* (ND = Not detected).
